# Evaluation of hand-assisted laparoscopic surgery of small intestinal neuroendocrine tumours as an alternative surgical treatment to open surgery

**DOI:** 10.1007/s00423-025-03658-z

**Published:** 2025-03-06

**Authors:** Branislav Klimácek, Tobias Åkerström, Matilda Annebäck, Per Hellman, Olov Norlén, Peter Stålberg

**Affiliations:** Department of Surgical Sciences, Uppsala University Hospital, 751 85 Uppsala, Sweden

**Keywords:** Hand-assisted laparoscopic surgery, Small intestinal neuroendocrine tumours, Open surgery, Radicality, Minimally invasive surgery

## Abstract

**Purpose:**

Small intestinal neuroendocrine tumours (SI-NETs) are the most common malignancy of the small bowel. Curative treatment is surgical, with exploratory laparotomy considered the standard approach. This study aimed to assess the outcomes of minimally invasive surgery compared to open approach for SI-NETs at the Endocrine surgical unit at Uppsala University Hospital.

**Methods:**

This retrospective cohort study included patients who underwent surgery for SI-NET between 2013 and 2023 at Uppsala University Hospital. Variables such as operative time, length of hospital stay, use of analgesia and radicality were compared between groups of patients operated on before and after 2019, when hand-port assisted laparoscopic surgery (HALS) for SI-NETs was introduced at our unit. Outcomes were further compared between open and hand-port assisted laparoscopic approaches. The primary outcome was the rate of radicality achieved for stage II-III patients. Secondary outcomes included operative time, the length of hospital stay and the use of epidural and patient-controlled analgesia.

**Results:**

Of 97 patients, 58 (59.8%) underwent open surgery and 39 (40.2%) underwent hand-port assisted laparoscopic surgery. There was no significant difference in operative time (121 min [91.3–150.3] vs 108 min [83–141]), length of hospital stay, 6 days [4–7] vs 5 days [4–8]), and surgical radicality in patients with stage II-III, 85.2% vs 100%, (*p* = 0.079). 86.2% of patients with explorative laparotomy required epidural analgesia compared to only 23.1% with HALS (*p *< 0.001).

**Conclusion:**

Hand-port assisted laparoscopic surgery of SI-NETs is a feasible approach that preserves radical resection while enhancing postoperative recovery, with a lower requirement of epidural analgesia.

## Introduction

Small intestinal neuroendocrine tumours (SI-NETs) are the most common malignancy of the small bowel [2, 3] arising from serotonin producing neuroendocrine cells [1, 7]. At diagnosis, about one third of all patients have multiple tumors [4–6], often identified only through diligent palpation intraoperatively. Even though small in size, a majority of newly diagnosed patients present with mesenteric lymph node metastasis (MLNMs) [7, 8]. Metastatic regional lymph node disease is accompanied by substantial mesenteric infiltration, which can result in excessive fibrotic overgrowth into the retroperitoneum, causing obstruction of the main mesenteric vessels, small intestine, duodenum and the ureters [9, 10]. Curative treatment of patients with SI-NETs is exclusively surgical with subsequent long survival [11–14].

While conventional laparoscopy is well-established for colorectal procedures, its feasibility in SI-NETs can be limited by technical challenges, such as the mobilization of the mesentery complicated by large metastatic lymph nodes accompanied by severe mesenteric fibrosis [15, 16] and the presence of multifocal primary tumors. In these situations, hand-assisted laparoscopic surgery (HALS) offers distinct advantages, including the ability to palpate tissues and guide dissection with surgeon´s hand. Moreover, HALS demonstrated excellent oncological outcomes with reduced operative time in malignancies like colorectal cancer [17]. The evidence supporting laparoscopic approaches for SI-NETs [18–22], however, remains sparse due to the rarity of the disease and its unique characteristics [23]. Consequently, minimally invasive surgery (MIS) has not yet become the standard approach for SI-NETs, unlike in other types of malignancies [3, 24].

The main objectives of this retrospective cohort study were to evaluate the outcomes of HALS of SI-NETs and assess its feasibility compared to the currently recommended explorative laparotomy.

## Material and methods

### Study design

This was a retrospective cohort study conducted at the endocrine surgical unit which is part of the ENETs Centre of Excellence at Uppsala University and Uppsala University Hospital.

The inpatient´s registry database at Uppsala University Hospital was searched using the International Classification of Diseases, Tenth Revision (ICD-10) code system, ICD code C17, C17.1 and C17.2. Patients above 18 years of age with a SI-NET diagnosis who underwent surgery for a primary SI-NET with or without metastasis at the Surgical Department/Endocrine surgical unit at Uppsala University Hospital between 2013 and 2023 were eligible for inclusion. Patients who had other simultaneous procedures performed or a tumour mass which was intraoperatively considered as unresectable; or had one or more prior major abdominal surgeries performed were excluded. Prior abdominal laparoscopic interventions and Cesarean sections were not defined as major. This method minimized bias in evaluating outcomes such as operative time and ensured that the comparison between the two surgical techniques remained as objective as possible.

The study was approved by the Swedish Ethical Review Authority (DNR 2011/375/1).

### Data sources

**Fig. 1:**
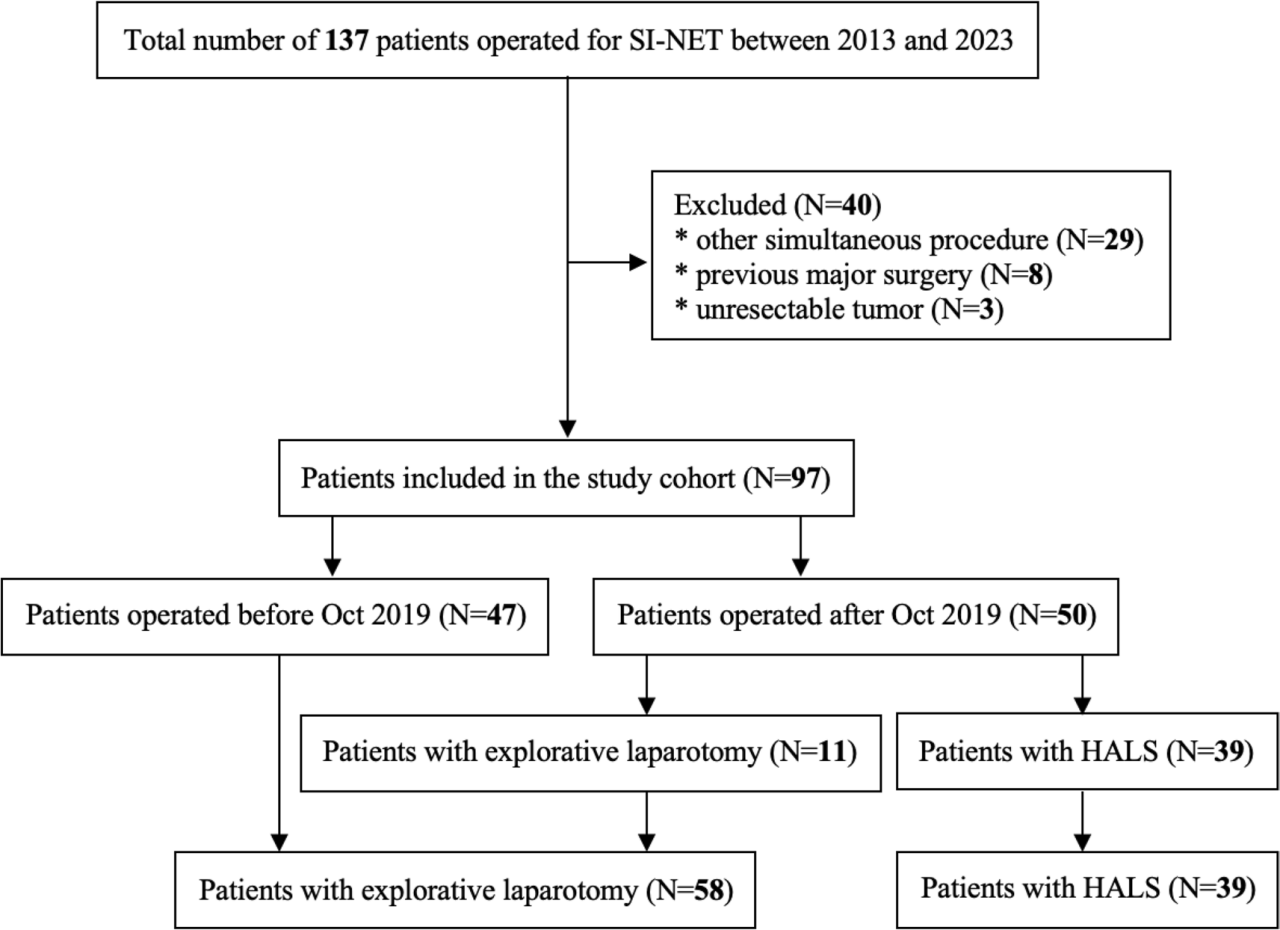
Patient flowchart

The medical charts of patients were reviewed for demographic, clinical, laboratory, histopathological data, as well as outcome variables for analysis. The outcome measures were defined as length of operative time, duration of hospital stay, necessity of epidural and patient-controlled analgesia, and radiologically confirmed radicality of surgery in stage II-III patients, for whom the surgery was deemed macroscopically radical intraoperatively and who underwent follow-up imaging. The radicality was defined as a disease-free state based on the first postoperative imaging with CT, MRI, or ^68^ Ga-DOTATOC-PET/CT. Primarily, individuals were separated into two groups based on exposure defined as the date of surgery, before or after October 8th 2019 when HALS for SI-NETs was implemented at the unit. In the initial years following the introduction of HALS, some patients continued to undergo open surgery, mostly by surgeons who lacked prior experience in HALS for other conditions. Comparing outcomes before and after October 2019 allowed for an assessment of whether the adoption of this technique improved overall patient outcomes across all eligible cases, regardless of the surgical approach used. This grouping minimized potential biases related to selection criteria during the initial transition phase. The data were further analysed by dividing them into groups based on surgical approach: 1. explorative laparotomy and 2. hand-assisted laparoscopic surgery. This division aimed to specifically evaluate the benefits and limitations of HALS in terms of operative outcomes, pain management, and recovery (Fig. 1).

### HALS technique

**Fig. 2:**
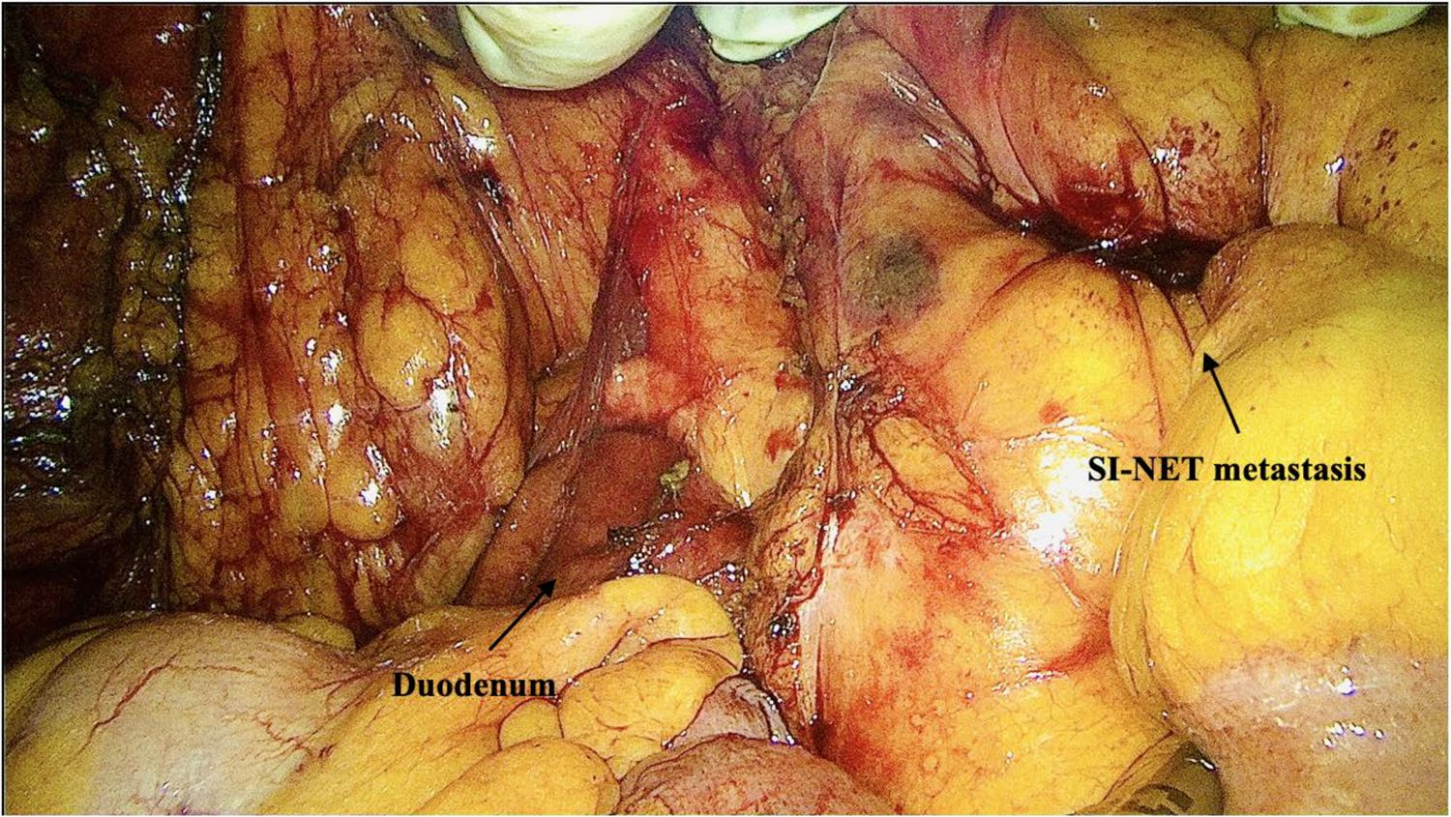
Mesenteric metastasis

The procedure started with a mini-laparotomy incision made in the midline above the umbilicus, with the incision length determined by the size of the surgeon´s hand (approximately 7 to 10 cm). An Alexis wound retractor (Applied Medical) was placed within the incision and two additional 12-mm trocars were positioned in the suprapubic region and in the lower left or right lower quadrants. To establish a pneumoperitoneum, the gel seal (Applied Medical) was fixed to the retractor at this stage. The surgeon´s non-dominant hand was then placed through the GelPort system enabling palpation, exposure, traction and dissection while the other hand controlled the laparoscopic instruments. The abdominal cavity, liver surface, diaphragms and pelvis were inspected using laparoscopy to identify unknown metastatic disease and the small intestine may be palpated from the ligament of Treitz distally to asses for multiple SI-NETs. Dissection started with mobilization of the terminal ileum from the retroperitoneum and continued along the white line of Toldt pushing the cecum and right colon medially and cranially, thereby defining the space between the colon/mesocolon and Gerota´s fascia up to the horizontal part of duodenum (Fig. 2). The right ureter was identified and freed to prevent any operative injury. At this stage, the intestine and mesentery were mobilized extracorporeally through the retractor, allowing for thorough palpation of the entire small bowel. Subsequently, metastatic lymph nodes dissection was performed alongside mesentery and bowel resection. The procedure was finalized with formation of either a hand-sewn or stapled enteroenterostomy or enterocolic anastomosis.

### Outcomes

Set primary outcome was the frequency of accomplished radicality after resection of SI-NETs and regional metastases, if present, with open surgery and HALS in patients with stage 2–3. Secondary outcomes included operative time, length of hospital stay and the frequency of administered analgesia in patients who underwent surgery with one of these techniques, irrespective of tumour stage.

### Statistical methods

Descriptive variables and outcome parameters are presented as median with interquartile range (Q1; Q3) and numbers (percentages), unless stated otherwise. The Mann–Whitney U test was used for continuous data, while the Chi-square test and Fisher´s exact tests were employed for nominal and ordinal data to compare the patient groups in the study. Statistical significance was defined as a p-value of < 0.05. A linear regression with scatter plot was used to estimate the correlation between operative time and the experience of the surgeon performing HALS procedure. All analyses were carried out using IBM SPSS Statistics.

## Results

A total of 137 patients were identified (Fig. 1). Twenty-nine patients were excluded as they had undergone another surgical procedure simultaneously, three had their tumour confirmed as unresectable intraoperatively; and eight were previously subjected to major abdominal surgery. In the end, ninety-seven patients were eligible for inclusion in the study. Two patients initially undergoing HALS (5.1%) required conversion to open surgery. To maintain consistency and reduce potential bias in outcomes evaluation, both cases were included in the data analysis for the minimal invasive surgery group.

### Analysis of Si-NET surgery before and after October 2019

Of the 97 patients, 47 were operated before October 2019 and 50 afterwards. There was no significant difference between these two groups in baseline variables, except Chromogranin A (CgA) and Ki-67 levels (Table 1, *p* = 0.007). The median age was 70.0 years (range 61–76) and 69.5 years (range 58.8–77) in the respective cohorts. In both groups a majority of patients were men, 34 (72.3%) vs 28 (56.0%). Approximately one fourth of patients had liver metastases, 25.5% and 24.0% respectively. No significant difference in disease stage of the groups were seen, Stage III (44.7% vs 48.0%) and IV (48.9 vs 50.0%). Abdominal pain and ileus were the most common indications for surgery in patients with Stage IV disease (74% vs 80.0%). All patients had Grade 1–2 tumours (Table 1). Four patients were excluded from the analysis due to the missing evaluation of Ki-67 on pathological examination. Epidural analgesia was administered significantly more in those operated prior to October 2019 than afterwards (89.4% vs 34.0%, *p*-value < 0.001). We observed statistically significant difference in operating time (122 min vs 106 min, *p*-value 0.038), but no in the length of post-operative stay, 5 days (6–7) vs 5 days (4–7.8). The radicality outcomes of surgery for stage II-III disease was similar in both groups (*p* = 0.255) with slight overlap of proportion confidence intervals (95% CI: 0.67 – 0.95) vs (95% CI: 0.8 – 0.99). Furthermore, the postoperative imaging modality (*p* = 0.127), was comparable between the two groups (Table 2).

**Table 1:** Baseline data in groups of Si-NET surgery before and after October 2019

Patient variabels	Si-NET surgery before 10/2019 *N* = 47	Si-NET surgery after 10/2019 *N* = 50	*p*-value
Age (years)	70 (61–76)	69.5 (58.8–77)	0.770*
Sex			0.094**
Female	13 (27.7)	22 (44.0)	
Male	34 (72.3)	28 (56.0)	
BMI	27.5 (25–31.3)	26 (23–32)	0.057*
ASA			0.345**
I	4 (8.5)	6 (12.0)	
II	31 (66.0)	26 (52.0)	
III	12 (25.5)	16 (32.0)	
IV	0 (0)	2 (4.0)	
Liver metastases	12 (25.5)	12 (24.0)	0.861**
CGA	5.7 (3–18.4)	3.3 (1.5–8.1)	0.007**
Stage			0.551**
I	0 (0)	0 (0)	
II	3 (6.4)	1 (2.0)	
III	21 (44.7)	24 (48.0)	
IV	23 (48.9)	25 (50.0)	
Multiple tumors (≥ 2)	26 (55.3)	18 (36.0)	0.077**
Tumor size (mm)	18 (12.5–22.5)	18 (14–22.6)	0.579*
Ki67%	1 (0.5–2)	1.3 (1–2)	0.007*
Grade			0.564***
I	38 (88.4)	41 (83.7)	
II	5 (11.6)	8 (16.3)	
Surgical indication St IV	***n* = 23**	***n* = 25**	0.608**
Abdominal pain/ileus	17 (74.0)	20 (80.0)	
GI-bleeding	1 (4.3)	2 (8.0)	
R0	5 (21.7)	3 (12.0)	

Values are median (Q1;Q3) and number (percent) unless otherwise stated. *CgA* Chromogranin

*ASA* American Society of Anesthesiologists physical status classification system. *BMI* Body mass index

^*^Mann–Whitney U test. **Chi square test. ***Ficher exact test

**Table 2:** Results in groups of Si-NET surgery before and after October 2019

Patient variables	Si-NET surgery before Oct/2019 *N* = 47	Si-NET surgery after Oct/2019 *N* = 50	*p*-value
Operative time (min)	122 (100–151)	106 (81.5–135)	0.038*
Epidural	42 (89.4)	17 (34.0)	< 0.001**
PCA	14 (29.8)	7 (14.0)	0.059**
Length of stay (days)	5 (6–7)	5 (4–7.8)	0.291*
Radicality of surgery St II-III	***n* = 22**	***n* = 24**	
	19 (86.4)	23 (95.8)	0.255**
Postoperative imaging method St II-III			
Ga68 PET CT	20 (83.3)	15 (60.0)	0.127**
CT	3 (12.5)	9 (36.0)	
MRI	0 (0)	1 (4.0)	
Missing value	1 (4.2)	0 (0)	

Values are median (Q1;Q3) and number (percent) unless otherwise stated

*PCA* Patient-controlled analgesia

^*^Mann–Whitney U test. **Chi square test

### Analysis of Explorative laparotomy and HALS

Characteristics of the patients in the explorative laparotomy (*n* = 58) and HALS (*n* = 39) group are presented in Table 3. We noted significantly higher CgA levels (5.7 mmol/l vs. 2,7 mmol/l, *p* = 0.003) and multifocality (56.9% vs. 28.2%, *p* = 0.012) in the exploratory laparotomy group. Tumour grade assessment in the pathological examination was absent in one patient and as result the patient was excluded from that analysis. For the remaining baseline variables, no significant differences were observed. Comparing outcome variables, the median operative time was not significantly different, 121 min (91.3–150.3) vs 108 min (83–141). Likewise, the length of hospital stay was similar, six days (4–7) vs five days (4–8). We noted a significant difference in the use of epidural analgesia, 86.2% vs 23.1% (*p*-value < 0.001). For patient-controlled anaesthesia (PCA) we noted a higher use of this in the exploratory laparotomy cohort although it did not reach statistical significance, 27.6% vs 12.8% (*p* = 0.083). There was no significant difference in surgical radicality in patients with stage II and III disease between surgical modalities (85.2% vs 100%, p 0.079) (Table 4).

**Table 3:** Baseline data for Laparotomy and HALS groups

Patient variables	Laparotomy *N* = 58	HALS *N* = 39	*p*-value
Age (years)	66 (60–76)	71 (60–77)	0.363*
Sex			
Female	19 (32.8)	16 (41.0)	0.406**
Male	39 (67.2)	23 (59.0)	
BMI	27 (23.5–31)	26 (23–29)	0.300*
ASA			
I	6 (10.3)	4 (10.3)	0.850**
II	36 (62.1)	21 (53.8)	
III	15 (25.9)	13 (33.3)	
IV	1 (1.7)	1 (2.6)	
Liver metastases	14 (24.1)	10 (25.6)	0.866**
CGA	5.7 (3–16)	2.7 (1.4–6.8)	0.003**
Stage			
I	0 (0)	0 (0)	0.791**
II	3 (5.2)	1 (2.6)	
III	26 (44.8)	19 (48.7)	
IV	29 (50.0)	19 (48.7)	
Multiple tumors (≥ 2)	33 (56.9)	11 (28.2)	0.012**
Tumor size (mm)	17 (13–22)	18.5 (14–23)	0.427*
Ki67%	1 (0.5–2)	1.45 (1–2)	0.068*
Grade			
I	46 (85.2)	33 (86.8)	1.000***
II	8 (14.8)	5 (13.2)	
Surgical indication in Stage IV	***n* = 29**	***n* = 19**	0.439**
Abdominal pain/ileus	22 (75.9)	15 (79.0)	
GI-bleeding	1 (3.4)	2 (10.5)	
R0	6 (20.7)	2 (10.5)	

Values are median (Q1;Q3) and number (percent) unless otherwise stated. *CgA* Chromogranin

^*^Mann–Whitney U test. **Chi square test. ***Ficher exact test

**Table 4:** Outcomes in Laparotomy and HALS groups

Patient variables	Laparotomy *N* = 58	HALS *N* = 39	*p*-value
Operative time (min)	121 (91.3–150.3)	108 (83–141)	0.189*
Epidural	50 (86.2)	9 (23.1)	< 0.001**
PCA	16 (27.6)	5 (12.8)	0.083**
Length of stay (days)	6 (4–7)	5 (4–8)	0.748*
Radicality of surgery St II-III	***n* = 27**	***n* = 19**	
	23 (85.2)	19 (100)	0.079**
Postoperative imaging method St II-III			
Ga68 PET CT	24 (82.8)	11 (55.0)	0.081**
CT	4 (13.8)	8 (40.0)	
MRI	0 (0)	1 (5.0)	
Missing value	1 (3.4)	0 (0)	

Values are median (Q1;Q3) and number (percent) unless otherwise stated

*PCA* patient-controlled analgesia

^*^Mann–Whitney U test. **Chi square test

**Fig. 3:**
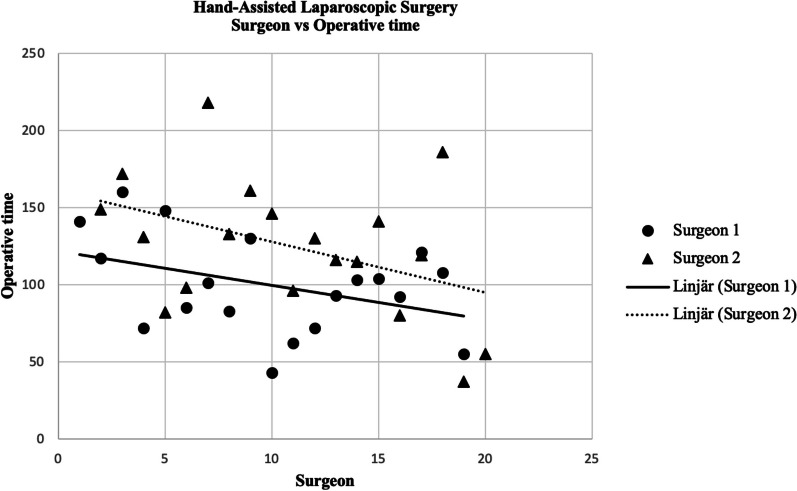
Scatter plot Operative time vs Surgeon

Additionally, the learning curve was assessed by comparing the operative times of HALS surgeries performed by experienced surgeons (defined as Surgeon 1 group), or those lacking previous experience in HALS technique (defined as Surgeon 2 group). The experienced surgeons were familiar with the HALS technique from prior use in distal pancreatic resections and splenectomies. The difference in operative time between two groups decreased by approximately 50% while both groups have seen improvement in surgical time (Fig. 3).

## Discussion

### Key results

The aim of this retrospective cohort study was to characterize and evaluate the role of HALS in the surgical management of patients diagnosed with SI-NETs compared to the conventional open approach. The results demonstrated that the radicality attained with this surgical technique was adequate and comparable to the common practice of open surgery. The absent use of epidural analgesia is commonly adopted in minimally invasive procedures. Given the uncertainty regarding the level of pain patients undergoing HALS for SI-NET would experience, epidural analgesia was administered mostly in the initial cases. However, the use of it was later avoided, as experience with the normal postoperative course after this procedure increased. Operative time and duration of hospital stay did not differ significantly between the two approaches. A shorter length of surgery is expected to be achieved as the HALS technique is further mastered across our unit. This trend is highlighted in the scatter plot (Fig. 3), which evaluates operative time in relation to surgeon proficiency. Although not examined in our study, the difference in abdominal incision length between laparotomy and HALS suggests that postoperative mobilization may be improved, and the risk of future incisional hernia reduced, with the latter approach.

Since October 2019, HALS has evolved to be the preferred technique at the Endocrine surgical unit at Uppsala University Hospital for all stage of SI-NET disease.

To date, few studies have explored the role of laparoscopic surgery in SI-NETs. Wang et al. [18] used a handport device to perform six successful laparoscopic resections in patients with stage IV disease, with an unknown primary tumour localization prior to surgery. Similarly, Reissman et al. [19] reported 15 patients who underwent surgery with laparoscopy and five HALS for SI-NET. In this study, radicality were not compromised. Notably, the mean operative time and length of hospital stay were also acceptable at 160 min and six days, respectively. Figueiredo et al. [20] reported a successful laparoscopic resection in 12 out of 73 patients with SI-NET. These patients required a significantly shorter hospital stay compared to a laparotomy cohort (six days vs eight days, *p* = 0.003). There was no difference in postoperative locoregional control between the groups. However, it must be emphasized that liver and lymph node metastases in the pre-operative assessment were less frequent in the laparoscopic group, 1 (8%) vs 42 (69%) and 3 (25%) vs 52 (85%), respectively.

One of the most recent studies on this subject was conducted by Kaçmaz et al. [21] with 34 patients, of whom 11 (33%) underwent open and 23 (67%) laparoscopic surgery. The surgical technique used was similar to the technique we describe. The only difference at our institution was that the surgeon used a hand-assisted approach intraabdominally, whilst carrying out resection of lymph node metastasis adjacent to the mesentery extracorporeally. The descriptive variables of the reported cohorts by Kaçmaz were also similar to those in the present study. For example, in the exploratory laparotomy cohort, stages III and IV accounted for 36% and 64%, of the participants respectively, compared with 55% vs 45% in the laparoscopic group, respectively. However, in contrast to our results, their analysis indicated significantly shorter median length of stay in the laparoscopic vs laparotomy group (4 days vs 8 days, *p* = 0.036), while there was no significant difference in administration of epidural analgesia between the groups. Interestingly, the operative time in laparoscopic surgery, although measured by different aggregator, was longer compared to HALS (mean 191 min vs median 117 min).

In another study, Kaçmaz et al. [22] assessed laparoscopic surgery for SI-NETs on a nationwide basis, which included 482 patients, with MIS performed in 140. The 5-year overall survival was significantly longer in the group with stage III disease who underwent laparoscopic resection, 84% compared to 71% for the open procedure (*p* = 0.004). However, the baseline characteristics of the laparotomy group demonstrated a higher clinical stage and amount of MLNMs. Operative time, the length of hospital stay and radicality/recurrence based on radiological follow-up were not appraised.

Our work has several limitations. Firstly, it is a retrospective study, which innately exposes it to selection bias. Our analysis was limited in its ability to account for various confounding variables, including the surgeons’ technical proficiency. This is particularly important, as HALS, like other laparoscopic and minimally invasive procedures, involves a corresponding learning curve that can significantly impact operative factors and outcomes, including operative time. Furthermore, the introduction of ^68^ Ga-DOTATOC/PET/CT and its preoperative diagnostic application may have contributed to more precise surgical procedures, potentially impacting the degree of surgical radicality.

The multifocality poses a significant challenge for achieving complete oncological resection. HALS provides an advantage by allowing manual palpation of the small bowel. Multifocality was observed more frequently in the laparotomy group compared to the HALS group (56.9% vs. 28.2%). This discrepancy remains unexplained, as bimanual palpation was conducted similarly in both approaches. It is important to note that these findings align with previously reported multifocality rates of 30–54% [4–6, 25, 26]. Notably, in our study, HALS achieved 100% radiological radicality postoperatively, demonstrating its reliability in managing of multifocality.

Although the single center design may limit generalizability, we are able to show that HALS for SI-NET is a feasible, surgically and oncologically safe procedure, which is easily adoptable from open laparotomy in a specialized center. Future work should be aimed at multicenter, international trials to achieve more reliable and generalizable results. To facilitate this, we would need to establish an accurate study protocol which includes quality assurance of surgical technique that remains consistent across study centres.

## Conclusion

For the particular indication of multifocal tumours, defined in most cases exclusively by palpation, and challenging resection of MLNMs with often substantial size, laparoscopy is not recommended by current guidelines. Since October 2019, HALS has evolved to be the preferred technique at the Endocrine surgical unit at Uppsala University Hospital for all stages of SI-NET disease. Based on the description and outcomes reported in the present study, HALS is a safe and effective technique in which all mandatory elements to secure oncological resection including the entire small bowel palpation for multiple primary tumours were executed as advised by the recommendations of European and American NET societies. We propose that a minimally invasive surgical approach, such as HALS, be considered for inclusion in the guidelines as a preferred alternative to explorative laparotomy for patients with SI-NET, regardless of clinical stage.

## Data Availability

No datasets were generated or analysed during the current study.
